# Noninvasive Multiparameter Monitoring for the Detection of Decompensated Heart Failure: Exploratory Study

**DOI:** 10.2196/59116

**Published:** 2025-10-08

**Authors:** Cyrille Herkert, Mayke van Leunen, Ignace Luc Johan De Lathauwer, Valerie Albertina Antonetta van Es, Jialu Tang, Aaqib Saeed, Rudolph Ferdinand Spee, Yuan Lu, Hareld Marijn Clemens Kemps

**Affiliations:** 1 Department of Cardiology, Máxima Medical Centre, Dominee Theodor Fliednerstraat 1, Eindhoven, 5631 BM, The Netherlands, 31408888000; 2 MoMiLab Research Unit, IMT School for Advanced Studies Lucca, Lucca, Italy; 3 Department of Industrial Design, Eindhoven University of Technology, Eindhoven, The Netherlands

**Keywords:** heart failure, remote patient monitoring, predictive model, photoplethysmography, accelerometry

## Abstract

**Background:**

Remote patient monitoring strategies in patients with heart failure (HF) are often based on manual readings and interpretation of various parameters by health care professionals. Automated multiparameter predictive models (MPMs) have the potential to improve early recognition of decompensated HF and to reduce the workload for both health care professionals and patients. To reduce costs and facilitate large-scale implementation, these models should preferably be based on noninvasive measurements, with user-friendly devices.

**Objective:**

This exploratory study aimed to evaluate whether an MPM, using various parameters from a wrist-worn device supplied with a photoplethysmography sensor and a triaxial accelerometer, contributes to the detection of decompensated HF and death in patients with unstable HF.

**Methods:**

Patients who were admitted to the hospital with acute decompensated HF, regardless of etiology or left ventricular ejection fraction, were instructed to wear a research-grade wrist-worn device from the moment of discharge. The device measured heart rate (HR), interbeat intervals (IBIs), respiration rate (RR), activity counts (AC), energy expenditure (EE), and sleep. Participants were instructed to wear the device 24 hours a day for 3 consecutive months. We evaluated 7 classifiers under four strategies for handling extreme class imbalance; the best model was then tested via leave-one-subject-out cross-validation on untouched data. The combined end point of interest was hospital readmission due to decompensated HF, decompensated HF treated at the outpatient clinic by increasing the loop diuretic dose, or death due to HF.

**Results:**

A total of 17 patients participated in the study (median age 77, IQR 70‐84 y; n=9, 53% male). During follow-up, the device-wearing compliance was 78% (55%‐81%). The activity-related parameters (EE and AC) performed best with respect to data quality: 72% and 79% of the data were of high quality, respectively. Concerning HR, 46% of the data were of high quality, whereas only 29% of the IBI and 14% of the RR data were of high quality. Sleep data were lacking 99% of the time during follow-up, resulting in exclusion from training the classifier. The most optimal model for the detection of the combined end point of HF deterioration showed a specificity of 97.2% and a sensitivity of 5.3% in the 2 weeks prior to an event (area under the curve=0.59) after leave-one-subject-out cross-validation analysis.

**Conclusions:**

An MPM using a noninvasive wrist-worn device, measuring HR, IBI, RR, AC, and EE, showed high specificity but low sensitivity for the prediction of decompensated HF and HF-related mortality. Low sensitivity likely reflects the extreme class imbalance and sequences with low data quality (especially HR, RR, and sleep), resulting in exclusion from training the MPM in our older, real-world HF cohort. Future studies should improve data fidelity and enroll larger cohorts to address class imbalance and enhance predictive performance.

## Introduction

Heart failure (HF) is a highly common clinical syndrome, affecting over 64 million people worldwide [1]. This amount is expected to increase by 46% between 2012 and 2030 [1,2], due to a combination of factors such as aging of the population and optimization of diagnostic and therapeutic management. Once being admitted to the hospital for decompensated HF, the 30-day all-cause readmission rate in patients with HF is up to 22% [3], accounting for the majority of the annual €25,000 (US $28,974) health care costs per patient with HF in the Western world [1].

In order to optimize HF care and to reduce the risk of readmission in this patient population, remote patient monitoring (RPM) strategies have been developed. It is generally conceived that monitoring the patients’ vital parameters and symptoms in the home environment enables early detection of deterioration, timely up-titration of HF medication, and improvement of self-management skills. Nevertheless, meta-analyses on noninvasive RPM show contradicting results [4-9]. Besides heterogeneity in study populations and end points, this may be explained by a large variety in sensor technology and (lack of) algorithms that were used.

In order to improve noninvasive sensor technology for the prediction of HF decompensation, it should comprise several characteristics. First, to improve the chances of preventing hospitalization by increasing the time frame to adjust medical therapy, sensors should detect signs of HF decompensation as early as possible. The first pathophysiological changes that lead to decompensated HF appear around 3 to 4 weeks before hospitalization due to decompensated HF, starting with an increase in cardiac filling pressures, followed by autonomic changes, and later, intrathoracic impedance changes [10]. Traditional RPM parameters, such as body weight or symptoms, change only a few days to a week before hospitalization [10]. Although previous research demonstrated that combining these traditional parameters into more complex, patient-tailored algorithms already increases the accuracy of predicting HF-related events [11,12], further improvement may be achieved by adding parameters that change earlier in the pathophysiological cascade of HF deterioration. As such, heart rate variability (HRV), a marker of autonomic adaptation, was shown to decrease at least 2 weeks prior to hospitalization [10,13]. Moreover, as the autonomic nervous system is involved in regulating the sleep-wake system [14], an increase in autonomic imbalance starting 2 weeks prior to hospital admission might alter sleep duration and patterns. Monitoring of sleep behavior might therefore also improve early detection of HF deterioration. Additionally, respiration rate (RR) increases as a result of fluid retention, but more importantly, sleep-related respiration patterns are altered as a result of autonomic imbalance [15]. Therefore, RR might also add value in the early detection of decompensated HF.

Second, current RPM programs mostly rely on daily spot measurements of several parameters. Continuous monitoring of parameters such as RR or heart rate (HR) might improve early prediction of decompensated HF, because it increases the ability to observe trends in these parameters.

Third, the use of a single device that continuously measures multiple parameters without the patient needing to take additional actions, rather than the use of multiple devices, might increase adherence to an RPM intervention, which attributes to its success. Whereas previous studies using multiparameter predictive models (MPMs) based on a single invasive device showed good diagnostic accuracy [16-18], their practical utility is limited due to the invasive nature of these devices. The LINK-HF study demonstrated that an MPM (including HR, HRV, arrhythmia burden, RR, gross activity, sleep, body tilt, and posture) using a chest patch could accurately predict the risk of rehospitalization for HF [19]. Wrist-worn devices, enabling monitoring of similar parameters, could add to this concept as they are widely accepted, can provide the patient with direct feedback, and are sustainable. To our knowledge, there are no studies available that evaluated an MPM to predict decompensated HF, based on different parameters obtained from a wrist-worn device.

This study aims to explore the predictive value of continuous data from a wrist-worn device (HR-related data, RR, activity, and sleep) to predict decompensated HF and death in older patients with unstable HF.

## Methods

### Study Protocol

This study aimed to develop an MPM, using various parameters from a wrist-worn device (Philips Data Logger [PDL]; Philips Electronics Nederland B.V.), for the detection of a combined end point: hospital readmission due to decompensated HF, decompensated HF treated at the outpatient clinic by increasing the dose of loop diuretics, or death due to HF. Patients who were admitted to the hospital with acute decompensated HF were asked for study participation. All participants were instructed to wear a PDL for a 3-month period after hospital discharge.

### Study Population

Adults (aged 18 y or older) with acute decompensated HF, who were admitted to the cardiology department of the Máxima Medical Center, Veldhoven, the Netherlands, were included in this study. Patients were included regardless of HF etiology or left ventricular ejection fraction (LVEF). Exclusion criteria were permanent atrial fibrillation (as sufficient data quality could not be assured in patients with permanent atrial fibrillation), not being able to perform daily physical activities (eg, due to an orthopedic or a neurological condition), not being able to wear the PDL due to a skin condition, and not having a Wi-Fi connection at home.

### Ethical Considerations

All patients provided written informed consent. The study was reviewed by the local medical ethics committee but received a waiver that medical ethics approval was not required. The study was registered at the Netherlands Trial Register (NL9038) and was performed in accordance with the principles of the Declaration of Helsinki.

### Philips Data Logger

**Figure 1.**
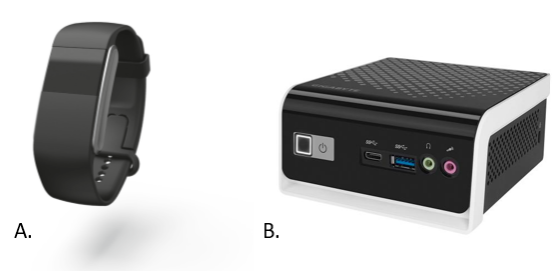
(A) Philips Data Logger. (B) Monitoring Study box.

The PDL is a wrist-worn, research-grade monitoring device measuring photoplethysmography (PPG)- and accelerometry-derived data (Figure 1A). Participants were blinded to their recorded parameters and did not receive feedback based on the measurements. The PDL is rain and splash resistant, but cannot be worn during showering or swimming. Data were offloaded from the PDL via Bluetooth as soon as the device was within reach of a gateway (Monitoring Study boX [MSX]; Figure 1B). The data were uploaded automatically from the MSX to the Philips Research cloud server via a 4G network. Parameters measured by the PDL that were used for analysis were: activity counts (AC), energy expenditure (EE), HR-related data such as HR and interbeat intervals (IBIs), RR, and sleep data (sleep stages and quality). All incoming data were classified as low, medium, or high quality by the software of Philips. This classification was used to evaluate the quality of the collected data over a 3-month period within this study population.

### Study Procedures

At hospital discharge (ie, in the recompensated state), the participants received a PDL together with an MSX. Instructions for use were given by the local investigators (CH, ML, and ILJL). Participants were asked to wear the PDL 24 hours per day (except when taking a shower or swimming), on the same wrist, for 3 consecutive months after hospital discharge. For every participant, the location of the PDL was registered (ie, right or left wrist), as well as the handedness of the participant. When data were lacking for a period of 3 days, the local investigators received a warning and contacted the participant to discuss and solve potential problems. During study participation, the local investigators periodically checked the participants’ medical records to report the events of interest, which were: a hospital readmission due to decompensated HF, an increase of loop diuretic dose at the outpatient clinic due to decompensated HF, and death, as expected to be the result of HF. An HF readmission is defined as a hospitalization resulting from an acute exacerbation of HF symptoms, primarily due to elevated ventricular filling pressures as a result of volume overload caused by the heart’s impaired ability to effectively pump blood [20]. The study was completed after the participant had worn the PDL for 3 months after hospital discharge, regardless of the encounter of a recurrent episode of decompensated HF.

### Data Preparation

In addition to the six physiological parameters collected by the PDL (AC, EE, HR, IBI, RR, and sleep), two extra parameters, related to wearing behavior, were included in the MPM. The first is “skin proximity,” a parameter calculated by Philips’ Software, revealing the distance between the skin and the device, varying from the device being worn properly to the device being off-skin. The second parameter, “fundamental,” indicates the proportion of the recorded data when the device was both powered on and properly worn to the recorded data of the entire period the device was powered on.

Eventually, all the collected parameters can be grouped as follows: (1) the parameters related to wearing behavior: skin proximity and fundamental; (2) the physiological parameters: AC, EE, HR, IBI, RR, and sleep stages and quality. In particular, HRV was calculated using the SD of all IBIs per 24 hours [21].

Only data segments labeled as high quality by the Philips software were included in the MPM. All data were split into 14-day segments, resulting in a total of 1218 time series. For each parameter time series, the mean, SD, minimum, and maximum values were extracted, yielding a total of 32 features used for model training.

These features were labeled into two distinct classes:

- Clinically stable, representing instances without the events of interest
- HF events, denoting instances with the events of interest (eg, decompensated HF treated at the outpatient clinic or leading to a hospital readmission, or death due to HF).

After labeling, the dataset consisted of 1209 instances of patients being clinically stable and 9 instances with HF events, indicating a substantial class imbalance.

### Multiparameter Predictive Model Training and Data Analysis

To address the extreme class imbalance in the dataset (1209:9, clinically stable:HF event), four modeling strategies were evaluated: baseline (unaltered data), random undersampling of the majority class, random oversampling of the minority class, and cost-sensitive learning that penalized false negatives more heavily. These strategies were applied across seven classifiers: decision tree, random forest (100 trees), gradient boosting (LogitBoost implementation), one-class support vector machine (RBF kernel), support vector classifier (RBF kernel), logistic regression (LASSO-regularized), and multilayer perceptron (single hidden layer with Rectified Linear Unit activation). Class weights or sampling adjustments were applied within each strategy depending on the model’s compatibility. Model training and selection were performed using stratified 5-fold cross-validation. The most promising model configuration was further validated using leave-one-subject-out cross-validation (LOOCV), ensuring that only real, unaltered data from the left-out participant was used for testing [22].

Since the included study population was diverse, including patients with different HF etiologies and LVEF, and the relatively small dataset, LOOCV allowed us to use as much data as possible for training the model. This method can reduce the bias and variance of the test error compared to using a single test set. With LOOCV, we anticipated learning how the predictive model performed for each patient and whether there were outlier participants. To evaluate the model’s performance, several key metrics were used, including the sensitivity, specificity, and area under the curve (AUC). Feature importance was evaluated using the out-of-bag permutation method for random forest, quantifying each feature’s contribution to model accuracy. Feature importances were aggregated by physiological modality (HR, IBI, RR, EE, AC, and wearing behavior) and normalized to 100% for interpretability.

Descriptive statistics were used to describe the baseline characteristics of the participants, the follow-up duration, and the occurrence of events.

**Figure 2.**
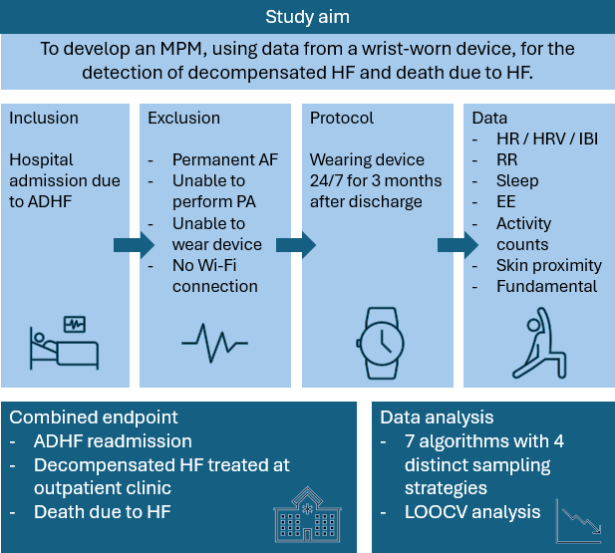
Overview of study aim and methods. ADHF: acute decompensated heart failure; AF: atrial fibrillation; EE: energy expenditure; HF: heart failure; HR: heart rate; HRV: heart rate variability; IBI: interbeat intervals; LOOCV: leave-one-out cross-validation; MPM: multiparameter prediction model; PA: physical activity; RR: respiration rate.

Mann-Whitney *U* tests were performed to analyze the differences in physiological parameters (HR, IBI, RR, EE, and AC) between time series during the stable phase and time series preceding an HF decompensation event. Time series analysis was performed to reveal trend changes in the physiological parameters over a 4-week period before a HF decompensation event. An overview of the study aims and methods is provided in Figure 2.

## Results

### Patient Characteristics

A total of 24 patients participated in the study; however, only 17 were included in the primary analysis. The reason for excluding seven participants was: one participant signed informed consent but withdrew from participation before hospital discharge; two participants experienced study participation as too demanding and decided to withdraw from participation shortly after inclusion; and in one participant, the data transfer was restricted due to technical problems, resulting in zero data transfer. Moreover, three additional patients were excluded from analysis, as they experienced an HF-related event (two hospital admissions due to decompensated HF and one HF decompensation treated at the outpatient clinic) within a week after inclusion and did not continue the study afterwards. As the aim was to observe trends in the data prior to an event, only patients with at least 2 weeks of data before a HF-related event were included. The patient characteristics of the remaining 17 participants are presented in Table 1.

**Table 1.** Patient characteristics.

Characteristics	Value (n=17)
Age (years), median (IQR)	77 (70‐84)
Sex, n (%)	
Male	9 (53)
Female	8 (47)
Height (m), median (IQR)	1.69 (1.61‐1.74)
Weight (kg), median (IQR)	80.6 (67.5‐94.5)
BMI (kg/m^2^), median (IQR)	30.7 (23.7‐32.3)
Race, n (%)	
Caucasian	17 (100)
Non-Caucasian	0 (0)
LVEF^a^ (%), median (IQR)	53 (43.5‐60.0)
HF^b^ type, n (%)	
HFrEF^c^	3 (17.6)
HFmrEF^d^	3 (17.6)
HFpEF^e^	11 (64.7)
Device therapy, n (%)	
Pacemaker	1 (6)
CRT(P/D)^f^	0 (0)
ICD^g^	0 (0)
Comorbidities, n (%)	
Paroxysmal AF^h^	9 (53)
Hypertension	11 (65)
Diabetes mellitus	7 (41)
Chronic kidney disease	5 (29)
Neurological history	5 (29)
Malignancy	4 (24)
Asthma or COPD^i^	2 (12)

a LVEF: left ventricular ejection fraction.

b HF: heart failure.

c HFrEF: heart failure with reduced ejection fraction.

d HFmrEF: heart failure with mildly reduced ejection fraction.

e HFpEF: heart failure with preserved ejection fraction.

f CRT(P/D): cardiac resynchronization therapy with either a pacemaker or a defibrillator.

g ICD: implantable cardioverter defibrillator.

h AF: atrial fibrillation.

i COPD: chronic obstructive pulmonary disease.

### Follow-Up and Events

The median follow-up duration of the 17 included participants was 92 (IQR 88‐93) days. Reasons for the premature ending of the study were: death (one participant), the start of palliative care resulting in the cancellation of further follow-up at the hospital (one participant), and skin irritation caused by the device (one participant). During follow-up, the compliance with wearing the device was 78% (55%‐81%). Nine events occurred during follow-up: seven times an increase of loop diuretic dose at the outpatient cardiology clinic due to decompensated HF, one hospital readmission due to decompensated HF, and one participant died due to worsening of HF. The median time from inclusion to the first event was 36 (IQR 22-76) days.

### Data Quality

The accelerometry-related parameters (EE and AC) performed best with respect to data quality: averaged per day, 72% and 79% of the data were of high quality, respectively. Concerning HR, 46% of the daily data were of high quality; 29% of the IBI (based on which HRV was calculated) data were of high quality. Only 14% of the RR data were of high quality, and 65% of this data were lacking. Sleep data were lacking 99% of the time during follow-up. Therefore, sleep features were excluded from the analysis due to nearly complete data absence.

### Performance Predictive Model

Table 2 presents the performance of 7 machine learning classifiers under four training strategies, evaluated using stratified 5-fold cross-validation. Ensemble models, particularly random forest and gradient boosting, achieved the highest AUC values (up to 0.99) when combined with oversampling or cost-sensitive training. Sensitivity ranged from 0% in baseline models such as logistic regression and multilayer perceptron to 96% in the best-performing model (random forest with oversampling), with an AUC of 0.96. Specificity remained high across most configurations, with values reaching up to 99.8%. One-class support vector machine and support vector classifier demonstrated limited sensitivity despite high specificity. These findings highlight the superiority of ensemble methods under imbalance-corrected training and the necessity of such strategies for improving predictive performance in small, event-sparse datasets.

**Table 2.** Comparative results of 5 different predictive models.

Strategy	Model	Sensitivity, mean (95% CI)	Specificity, mean (95% CI)	AUC^a^, mean (95% CI)	NPV^b^, mean (95% CI)	PPV^c^, mean (95% CI)
Baseline	DecisionTree	7.14 (4.28-10.00)	98.1 (96.7-99.5)	0.50 (0.45-0.55)	99.59 (94.61-100)	1.7 (0.0-6.14)
Baseline	RandomForest	51.43 (47.86-55.00)	95.0 (94.0-96.0)	0.70 (0.66-0.74)	99.73 (94.74-100)	10.0 (9.0-11.0)
Baseline	GradientBoosting	55.0 (45.5-64.5)	98.0 (97.0-99.0)	0.72 (0.67-0.77)	99.73 (94.74-100)	10.0 (9.0-11.0)
Baseline	OneClassSVM	0.0 (0.0-0.0)	70.0 (69.0-71.0)	0.67 (0.62-0.72)	99.05 (94.10-100)	0.0 (0.0-0.0)
Baseline	MLP^d^	0.0 (0.0-0.0)	63.0 (62.0-64.0)	0.6 (0.57-0.63)	99.05 (94.10-100)	0.0 (0.0-0.0)
Baseline	LR^e^	0.0 (0.0-0.0)	63.0 (62.0-64.0)	0.6 (0.57-0.63)	99.05 (94.10-100)	0.0 (0.0-0.0)
Baseline	SVC^f^	0.0 (0.0-0.0)	80.0 (79.0-81.0)	0.98(0.93-1.00)	99.05 (94.10-100)	0.0 (0.0-0.0)
Undersample	DecisionTree	99.0 (98.0-100.0)	37.81 (35.92-39.70)	0.74 (0.70-0.78)	99.0 (98.0-100.0)	1.52 (1.44-1.60)
Undersample	RandomForest	99.0 (98.0-100.0)	43.97 (41.77-46.17)	0.91 (0.86-0.96)	99.0 (98.0-100.0)	1.68 (1.60-1.76)
Undersample	GradientBoosting	99.0 (98.0-100.0)	35.41 (33.64-37.18)	0.9 (0.85-0.95)	99.0 (88.0-100.0)	1.46 (1.39-1.53)
Undersample	OneClassSVM^g^	99.0 (98.0-100.0)	91.92 (87.32-96.52)	0.93 (0.88-0.98)	99.0 (98.0-100.0)	10.61 (10.08-11.14)
Undersample	MLP	0.0 (0.0-0.0)	99.0 (98.0-100.0)	0.6 (0.57-0.63)	99.05 (94.10-100.0)	0.0 (0.0-0.0)
Undersample	LR	0.0 (0.0-0.0)	99.0 (98.0-100.0)	0.6 (0.57-0.63)	99.05 (94.10-100)	0.0 (0.0-0.0)
Undersample	SVC	92.86 (88.22-97.50)	58.63 (55.70-61.56)	0.83 (0.79-0.87)	99.88 (94.89-100)	2.11 (2.00-2.22)
Oversample	DecisionTree	92.0 (88.5-95.5)	89.5 (85.3-93.7)	0.94 (0.90-0.98)	98.7 (97.6-99.8)	7.8 (5.5-10.1)
Oversample	RandomForest	96.0 (93.8-98.2)	91.8 (87.9-95.7)	0.96 (0.93-0.99)	99.2 (98.3-100)	11.3 (9.2-13.4)
Oversample	GradientBoosting	94.5 (91.6-97.4)	91.0 (86.9-95.1)	0.95 (0.92-0.98)	99.0 (98.0-100)	10.2 (8.2-12.2)
Oversample	OneClassSVM	88.0 (84.0-92.0)	85.5 (80.5-90.5)	0.89 (0.85-0.93)	97.8 (96.4-99.2)	6.4 (3.9-8.9)
Oversample	MLP	33.5 (27.5-39.5)	90.5 (86.2-94.8)	0.66 (0.62-0.70)	95.4 (92.9-97.9)	2.9 (1.3-4.5)
Oversample	LR	0.0 (0.0-0.0)	99.52 (94.54-100)	0.65 (0.62-0.68)	99.05 (94.10-100)	0.0 (0.0-0.0)
Oversample	SVC	85.0 (80.8-89.2)	88.2 (83.4-93.0)	0.90 (0.87-0.93)	97.2 (95.5-98.9)	5.6 (3.4-7.8)
CostSensitive	DecisionTree	64.29 (61.08-67.50)	99.8 (94.81-100)	0.98 (0.93-1.00)	99.66 (94.68-100)	5.0 (1.25-8.75)
CostSensitive	RandomForest	71.43 (67.86-75.00)	99.0 (98.0-100)	0.99 (0.94-1.00)	99.73 (94.74-100)	9.0 (8.0-10.0)
CostSensitive	GradientBoosting	71.43 (67.86-75.00)	99.0 (98.0-100)	0.99 (0.94-1.00)	99.73 (94.74-100)	9.0 (8.0-10.0)
CostSensitive	OneClassSVM	0.0 (0.0-0.0)	99.0 (98.0-100)	0.97 (0.92-1.00)	99.05 (94.10-100)	0.0 (0.0-0.0)
CostSensitive	MLP	0.0 (0.0-0.0)	98.29 (93.38-100)	0.59 (0.56-0.62)	99.03 (94.08-100)	0.0 (0.0-0.0)
CostSensitive	LR	0.0 (0.0-0.0)	99.0 (98.0-100)	0.6 (0.57-0.63)	99.05 (94.10-100)	0.0 (0.0-0.0)
CostSensitive	SVC	0.0 (0.0-0.0)	99.0 (98.0-100)	0.97 (0.92-1.00)	99.05 (94.10-100)	0.0 (0.0-0.0)

a AUC: area under the curve.

b NPV: negative predictive value.

c PPV: positive predictive value.

d MLP: multilayer perceptron.

e LR: logistic regression.

f SVC: support vector classifier.

g SVM: support vector machine.

**Figure 3.**
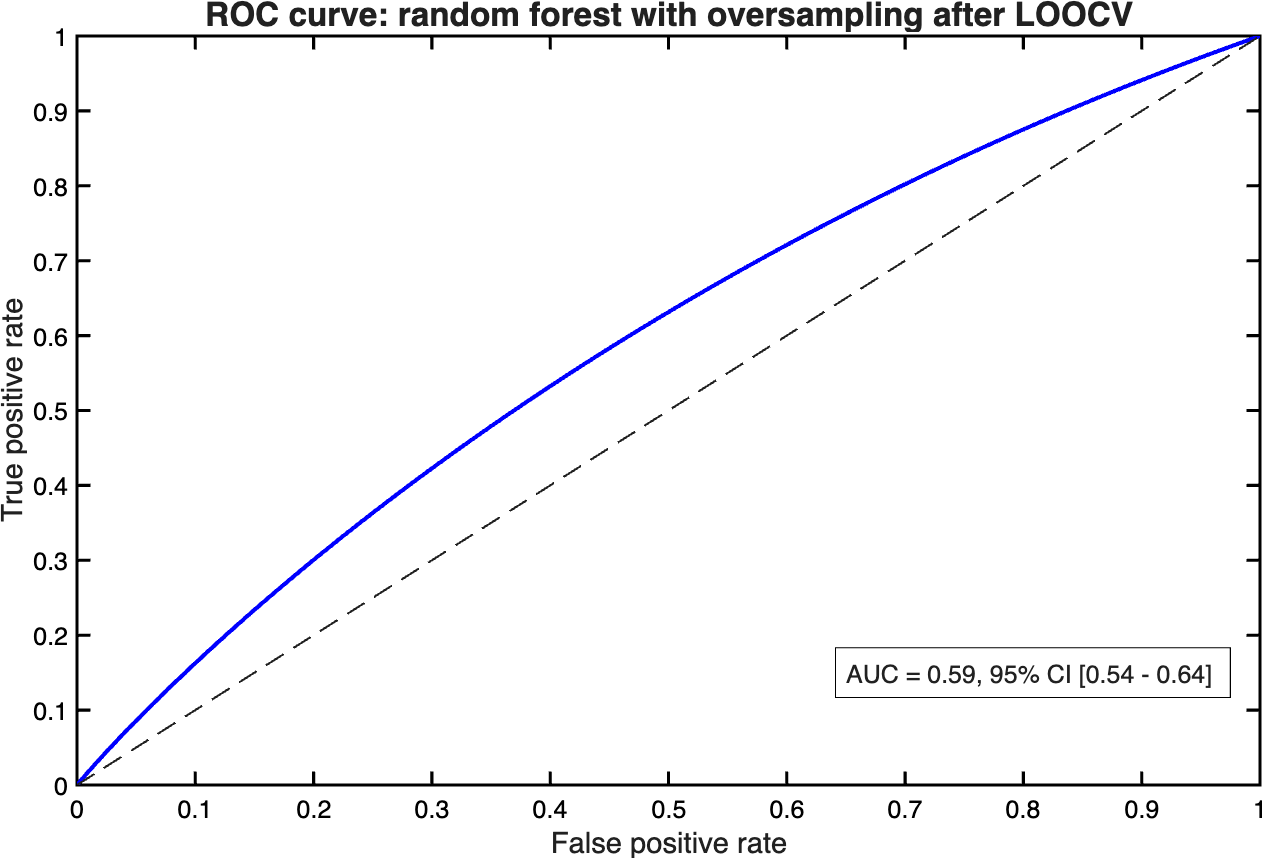
ROC curve for the random forest model with oversampling, evaluated by LOOCV. The solid blue line shows model performance across all thresholds, the dashed black diagonal represents chance level (AUC=0.5), and the text box reports the AUC (0.59, 95% CI 0.54-0.64). AUC: area under the curve; LOOCV: leave-one-subject-out cross-validation; ROC: receiver operating characteristic

LOOCV was applied using the best-performing model configuration (random forest with oversampling) to assess generalizability across individual participants. As anticipated, performance metrics were lower compared to stratified 5-fold cross-validation, reflecting the impact of increased variance and the challenge of predicting rare events in single-subject test folds. Pulled AUC across participants was 0.59(95% CI 0.54-0.64; Figure 3), with individual values ranging from 0.47 to 0.64. Specificity remained consistently high (97.2%, 95% CI 95.9-98.5), whereas sensitivity was notably limited (5.3%, 95% CI 0.0-15.5), underscoring the difficulty of reliably identifying decompensation events in highly imbalanced datasets (Table 3).

**Table 3.** Iterations of the LOOCV analysis.

Participant number	AUC^c^ (95% CI)	Specificity (95% CI)	Sensitivity (95% CI)
1	0.61 (0.56-0.66)	97.1 (95.8-98.4)	6.2 (0.0-17.4)
2	0.57 (0.51-0.63)	96.5 (94.5-98.5)	4.3 (0.0-14.8)
3	0.59 (0.55-0.63)	98.3 (97.2-99.4)	5.1 (0.0-15.1)
4	0.55 (0.52-0.58)	95.9 (94.4-97.4)	3.9 (0.0-13.3)
5	0.60 (0.55-0.65)	97.8 (96.6-99.0)	7.1 (0.0-18.1)
6	0.58 (0.52-0.64)	96.7 (95.3-98.1)	4.9 (0.0-15.7)
7	0.62 (0.57-0.67)	97.9 (96.8-99.0)	6.8 (0.0-17.7)
8	0.48 (0.47-0.49)	98.5 (97.7-99.3)	0.0 (0.0-0.0)
9	0.64 (0.60-0.68)	98.1 (97.1-99.1)	8.7 (0.0-20.0)
10	0.53 (0.49-0.57)	96.0 (94.0-98.0)	3.1 (0.0-12.6)
11	0.58 (0.53-0.63)	96.8 (95.5-98.1)	5.4 (0.0-15.8)
12	0.59 (0.54-0.64)	97.5 (96.3-98.7)	5.9 (0.0-16.1)
13	0.61 (0.56-0.66)	97.6 (96.6-98.6)	6.5 (0.0-17.1)
14	0.56 (0.51-0.61)	97.2 (95.9-98.5)	4.4 (0.0-14.2)
15	0.57 (0.52-0.62)	97.3 (96.2-98.4)	4.6 (0.0-14.5)
16	0.56 (0.51-0.61)	96.9 (95.7-98.1)	4.2 (0.0-13.9)
17	0.59 (0.54-0.64)	97.4 (96.3-98.5)	5.8 (0.0-15.8)
All participants	0.59 (0.54-0.64)	97.2 (95.9-98.5)	5.3 (0.0-15.5)

a LOOCV: leave-one-subject-out cross-validation.

b The table shows the AUC, specificity, and sensitivity when (eg, in row 1) participant 1 is left out of the analysis.

c AUC: area under the curve.

Feature importance analysis indicated that HR metrics contributed most to the model’s predictions (36%), followed by IBIs (18%), AC (14%), RR (12%), and EE (11%). Wearing behavior (9%) also contributed modestly.

### Trends in Physiological Parameters

Table 4 represents the mean and SD of the physiological parameters for both the stable phase and the phase preceding HF decompensation. Notably, 2 weeks prior to the HF decompensation event, HR significantly increased by 11 beats per minute. Additionally, the daily IBI, related to the HRV index, decreased significantly by 124 ms. All other parameters showed no significant differences.

**Figure 4.**
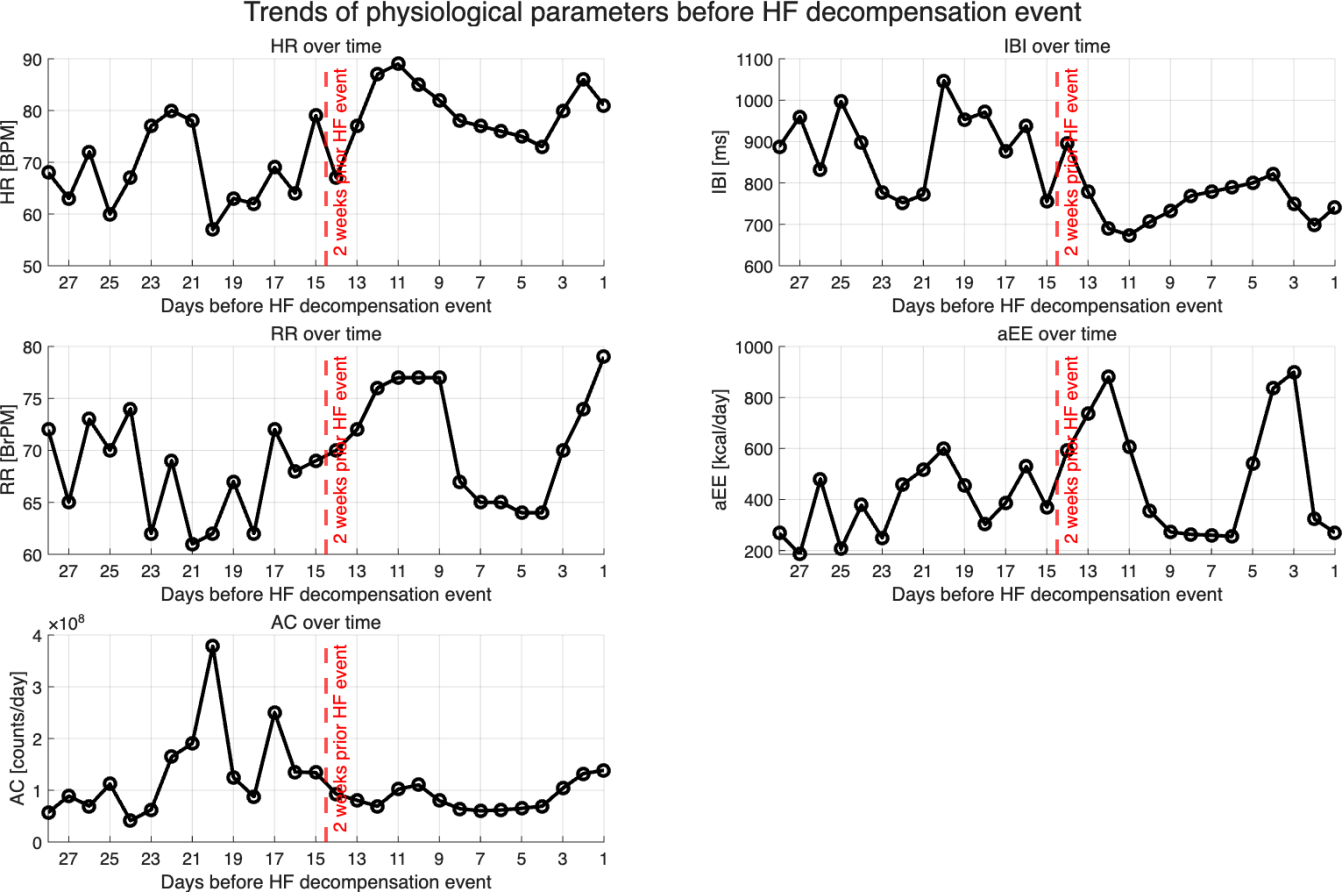
Timeseries of the physiological parameters 4 weeks prior to HF decompensation events with the daily average values of the HR, IBI, RR, EE, and AC of an example patient. AC: activity count; EE: energy expenditure; HF: heart failure; HR: heart rate; IBI: interbeat interval; RR: respiration rate.

The time series analysis (Figure 4) also revealed a trend in the physiological parameters over the 4-week period before the HF decompensation event. HR demonstrated an upward trajectory, while IBI displayed a downward trend, indicating an imbalance in autonomic regulation.

**Table 4.** Means and SDs of physiological parameters during the stable phase and decompensated heart failure phase.

	Stable phase, mean (SD)	ADHF^a^ phase, mean (SD)	Δ	*P* value
HR^b^ (bpm)	68 (7)	79 (7)	11 (0)	.03
IBI^c^ (ms)	886 (91)	762 (60)	−124 (30)	.03
RR^d^ (BrPM)	67 (8)	71 (6)	4 (2)	.22
EE^e^ (kcal/day)	375 (129)	466 (304)	91 (175)	.69
AC^f^ (counts/day)	13e+10 (9e+7)	85e+10 (3e+7)	72e+10 (6e+7)	.29

a ADHF: acute decompensated heart failure.

b HR: heart rate.

c IBI: interbeat interval.

d RR: respiratory rate.

e EE: energy expenditure.

f AC: activity counts.

## Discussion

### Principal Findings

This exploratory study is one of the first that used noninvasive continuous data monitoring (rather than spot measurements) to predict HF-related events in an older HF population in a real-life setting. An MPM based on HR, IBI, RR, AC, and EE predicted the combined end point of decompensated HF and death with a high specificity but low sensitivity. This limited sensitivity likely reflects two key challenges: first, the poor signal quality observed from wrist-worn sensors in an older cohort, which resulted in the removal of data from several 14-day sequences; and second, the severe imbalance between stable-phase and decompensation events, which makes it difficult for even powerful classifiers to learn a reliable decision boundary without overfitting. Future work should therefore focus both on boosting the fidelity of wearable-device data in real-world, older populations and aiming to increase the number of monitored events—either by extending follow-up or broadening inclusion criteria to mitigate extreme class imbalance.

### HR-Related Data (HR and IBI) and RR

HR-related parameters (HR and IBI) were the most important contributors to the prediction model. These results are in line with previous studies showing that an increased HR is associated with a higher risk of hospital readmissions and mortality in stable chronic patients with HF with sinus rhythm, regardless of LVEF [23,24]. Moreover, the amount of evidence is growing that a higher HR at discharge after an episode of acute decompensated HF is associated with a worse prognosis [25-27]. When HR is followed over time, an increase in HR is associated with a worse prognosis and higher risk of hospital readmission [18,23]. In addition, previous studies in patients with implantable devices showed that HRV declines prior to HF decompensation as a result of autonomic imbalance [10,18]. In patients with HF with preserved ejection fraction (HFpEF), less is known about the prognostic role of HRV, although a reduced HRV seems to predict 1-year mortality in these patients [28]. To our knowledge, there is no data available on longitudinal follow-up of HRV in patients with HFpEF prior to an episode of decompensated HF. Nevertheless, as an increased autonomic imbalance precedes congestion [29], HRV is likely to change earlier in the cascade than traditional RPM parameters such as weight and symptoms, and should therefore be considered in future RPM studies. RR was the fourth most important contributor in the prediction model in this study. Previous research on RR data extracted from implantable cardioverter defibrillators/cardiac resynchronization therapy devices in New York Heart Association class III patients with HF showed that median, minimum, and maximum RR were significantly elevated prior to HF decompensation [30]. Nevertheless, in patients with HF with concomitant sleep-disordered breathing treated with adaptive servo-ventilation, nightly RR was significantly lower, and apnea-hypopnea index was significantly increased in patients with decompensated HF [31]. This indicates that the change in RR pattern is dependent on the presence of comorbidities and the timing of measurement. Studies about the accuracy of wrist-worn devices in the estimation of RR are scarce. A validation study in healthy individuals, in resting conditions, showed that a wrist-worn PPG-based device could accurately measure RR [32]. Moreover, a previous validation study with the Philips health watch showed that it was able to estimate RR in healthy individuals undergoing a laboratory protocol with sufficient accuracy [33]. Nightly RR in individuals undergoing polysomnography can also be accurately measured by a consumer wrist-worn device; however, accuracy is decreasing with worsening of sleep apnea [34]. In addition, in patients undergoing postabdominal surgery residing at the intensive care or postanesthesia unit, RR can be measured sufficiently accurately using a PPG wristband, although the detection of RR was easily corrupted by motion artifacts, leading to the exclusion of 66% of the measurements [35]. This highlights the need for validation of wrist-worn devices in the HF population in free-living conditions. The limited amount of high-quality RR data in this study might partially be attributed to external factors such as motion artifacts in free-living conditions and closeness of the device to the skin [36]. Individual factors, such as age or BMI, can also contribute to noise in the PPG signal. Particularly, in older people, the PPG signal may be negatively influenced by decreased arterial compliance and thinning of the skin [36]. Moreover, the BMI in our study population was high, which is associated with an increased cutaneous blood flow, a decreased capillary density, and an increased transepidermal water loss. These BMI-induced changes negatively affect the PPG signal [36].

### Activity Monitoring

Activity-related parameters (AC and EE) also contributed to the best predictive model. This is consistent with a previous systematic review, which showed that noninvasive actigraphy-quantified physical activity showed a strong inverse relationship with intercurrent HF events such as hospitalizations [37]. However, most of these studies used a single physical activity measurement to quantify the level of physical activity in relation to the risk of HF-related events. Cakmak et al [38] showed that continuous, smartphone-based monitoring of accelerometry data, alone or together with other parameters, is helpful in predicting HF decompensation up to 6 days in advance of the encounter of the event. Moreover, Adamson et al [13] showed that invasively monitored physical activity, using cardiac resynchronization therapy data, significantly decreased prior to a HF hospitalization, although these changes were less sensitive than changes in HRV measured with the same device. The SELENE-HF study also used invasively monitored physical activity in an MPM to predict hospitalization in patients with HF, although its contribution to the model was relatively low as compared to HR [18]. An explanation for the limited predictive value of activity behavior in this study may be related to data quality and accuracy. Although a previous validation study with a Philips health watch showed that it accurately estimates EE in healthy people undergoing a laboratory protocol [33], these results might not be applicable to older patients with HF in free-living conditions. Indeed, we recently showed that the accuracy of commercially available wrist-worn devices in estimating EE in patients with HF performing a laboratory protocol was low [39]. More importantly, the responsiveness, that is, the ability to detect within-patient changes in EE within patients, was also poor [39]. This indicates the need for population-specific algorithms for monitoring activity in the home environment. Moreover, it was shown that hip placement of an accelerometer led to a more accurate estimation of metabolic equivalents as compared to wrist, ankle, upper arm, and thigh placement, in older adults performing a laboratory protocol [40]. To our knowledge, it is not known to what extent the accelerometer location is relevant in older patients with HF in free-living conditions.

### Sleep Monitoring

Data on sleep stages and quality could not be accurately measured by the device used in this study, leading to the exclusion of sleep features from our prediction model. For this reason, sleep data were not included in the MPM. Nevertheless, sleep data are expected to be of particular value in future prediction models, as previous research has found an association between delayed bedtime or wake-up time and the development of congestive HF [41]. Moreover, congestive HF negatively influences sleep quality [42,43]. As decompensated HF is often associated with orthopnea and nycturia, sleep duration and quality are expected to decrease upon fluid retention. Additionally, 50%‐75% of the patients with HF have concomitant sleep-disordered breathing [44], which can worsen during a HF exacerbation. This also contributes to poorer sleep quality during the night and somnolence during the day. However, it remains questionable how to accurately assess sleep data in patients with HF in the home environment using a patient-friendly device. Although commercially available sleep tracking devices tend to accurately detect sleep, they detect wake less accurately as compared to polysomnography in healthy individuals, whereas sleep stage assessments were inconsistent as well [45,46]. In the study of Chinoy et al [45], commercially available sleep trackers outperformed research-grade actigraphy devices, and notably, all devices performed worse during nights with disrupted or poor sleep. Other nonwrist-worn sleep tracking devices showed variable agreement with polysomnography in individuals without uncontrolled respiratory conditions [47]. To our knowledge, sleep-tracking devices have not been specifically validated in an HF population in which interrupted sleep is highly common. Therefore, more research is needed in this specific population.

### Future Implications

#### PPG Signal-to-Noise Ratio

Although the individual parameters used in the algorithm (HR, IBI, AC, RR, and EE) are potentially indicative of HF decompensation, the combination of these parameters led to a model with high specificity but low sensitivity. This indicates that while the model is reliable in identifying clinically stable periods (ie, few false alarms), it fails to detect the majority of actual HF decompensation events. In other words, when an alarm is generated by the MPM, decompensated HF (or death due to HF) will likely occur in the 2-week period, but many events occur without any alarm being raised. Due to this high miss rate, the current model is not suitable for use in daily clinical practice. To further improve the prediction model, several issues should be addressed. First, there were far more “stable” data points than decompensation events, and even with oversampling and cost-sensitive methods, the model struggled to distinguish the rare events without overfitting to the majority class. To address this, future studies should increase the number of decompensation cases, either by extending the monitoring period, increasing the patient population, or stricter inclusion criteria, to balance the dataset at the source. Second, the question is whether the device used in this study provides accurate measurements in older patients with HF in free-living conditions and whether it accurately detects intraindividual changes. As patients with HF often have a disturbed respiration pattern, a disturbed sleep pattern, function in the low-intensity physical domain, and have ectopic heartbeats or intermittent heart rhythm disturbances, validation results of wrist-worn devices in healthy individuals cannot be extrapolated to the HF population. Ideally, the device should be first validated in the HF population, and population-specific algorithms should be developed. Third, several factors negatively influence the wrist-based PPG signal, such as closeness of the device to the skin, skin properties, and vascular phenomena related to older age. Therefore, other types of non-PPG devices, such as the thoracic patch, using a combination of electrocardiogram and impedance, which was used in the LINK-HF study [19], might deliver more accurate results when firmly attached to the skin. Wearing a patch might also lead to a higher adherence as compared to a wrist-worn device, as wrist-worn devices can easily be removed and forgotten afterwards. In a real-world setting, the expected adherence to a wrist-worn device might be even lower than was reported in this study, as in this study, the local investigators contacted participants when data were lacking for 3 consecutive days. Nevertheless, patches also have disadvantages as they are often disposable and might be costly when they have to be replaced repeatedly. In addition, as the skin of older people is thinner and easily disrupted, patches might lead to skin problems when prolonged wearing is necessary. Moreover, wrist-worn devices are generally accepted, also in the older population, and provide an opportunity for direct feedback based on the measured parameters. Moreover, wrist-worn devices are far less costly (ie, an estimated €150 [US $176]) as compared to invasive devices and are expected to be suitable for reuse. Whether they are also less costly as compared to other noninvasive devices should, however, be further evaluated. Wrist-worn devices with a higher signal-to-noise ratio that are tailored to an older population might therefore be preferred over other devices.

#### Workflow Methodologies

In order to obtain a greater amount of high-quality data, some alterations in the workflow may be considered. For example, asking the patient to perform daily recordings lasting a few minutes while sitting completely still. This might lead to a higher amount of high-quality data, which might improve the predictive value of the algorithm, although this hypothesis should be tested. High-quality sleep and activity data cannot be obtained using this strategy. Using a strap that connects the PPG sensor closer and more stable to the skin might lead to a higher data quality in all categories (ie, HR, HRV, RR, physical activity, and sleep). This concept, however, needs to be developed and tested before implementation.

#### Potential of Artificial Intelligence

Although the models that have been evaluated in this study did not lead to one with sufficient diagnostic accuracy, artificial intelligence has great potential to increase the efficiency of health care, particularly in the prediction of worsening of HF. Machine-learning and deep learning methods based on noninvasive collected parameters do show promising results in the early detection of decompensated HF, although the effects on clinical outcomes should be further evaluated [48,49].

### Strengths and Limitations

This study is one of the first to develop and evaluate an MPM for the early detection of worsening HF, using a single, noninvasive, wrist-worn device. A major limitation of this study is the inclusion of a limited number of participants. Including more participants in the study, that is, increasing the dataset size, might have improved the model’s performance and class balance. In this study, only nine events occurred, of which seven were episodes of decompensated HF treated at the outpatient clinic. Only one participant was readmitted to the hospital due to decompensated HF. Another limitation was that the included population predominantly consisted of patients with HFpEF, and patients with permanent atrial fibrillation were excluded from study participation. Therefore, the results cannot be extrapolated to the entire HF population. Nevertheless, being an exploratory study, the results of this study have shed light on the future steps needed to optimize the use of wrist-worn wearables to predict HF events in older patients.

### Conclusions

A noninvasive MPM using a single wrist-worn device measuring HR, IBI (HRV), RR, AC, and EE showed a high specificity but a low sensitivity in predicting decompensated HF and death. An important reason for the low sensitivity could be the extreme class imbalance and low data quality (specifically in IBI, RR, and sleep data), resulting in the loss of usable input for the MPM in this real-world HF population. Future studies are needed for validation and optimization of sensor technology in wrist-worn devices for this specific population.

## Abbreviations

AC: activity counts
AUC: area under the curve
EE: energy expenditure
HF: heart failure
HFpEF: heart failure with preserved ejection fraction
HR: heart rate
HRV: heart rate variability
IBI: interbeat interval
LOOCV: leave-one-out cross-validation
LVEF: left ventricular ejection fraction
MPM: multiparameter predictive model
MSX: monitoring study box
PDL: Philips data logger
PPG: photoplethysmography
RPM: remote patient monitoring
RR: respiration rate
